# Case Report: Supraorbital keyhole resection of IDH-mutant grade 3 astrocytoma of the medial orbitofrontal region

**DOI:** 10.3389/fonc.2026.1813949

**Published:** 2026-07-10

**Authors:** Nurali Ashirov, David Pochivalov, Daniyar Zhamoldin, Viktor Aleinikov, Berik Zhetpisbayev, Aisa Nurpeisov, Vladimir Kenzhebayev, Serik Akshulakov

**Affiliations:** 1 Department of Minimally Invasive Neurosurgery, JSC National Centre for Neurosurgery, Astana, Kazakhstan; 2 Scientific Research Management Department, JSC National Centre for Neurosurgery, Astana, Kazakhstan; 3 Department of Pathology, JSC National Centre for Neurosurgery, Astana, Kazakhstan; 4 JSC National Centre for Neurosurgery, Astana, Kazakhstan

**Keywords:** astrocytoma, high-grade glioma, IDH-mutant astrocytoma, supraorbital eyebrow approach, keyhole surgery, minimally invasive neurosurgery, orbitofrontal tumor, cosmetic outcomes

## Abstract

**Background:**

High-grade gliomas located in the medial orbitofrontal region present significant surgical challenges due to their deep location and proximity to critical neurovascular structures. While the supraorbital eyebrow approach is widely used for extra-axial skull base lesions, its application to intra-axial high-grade gliomas remains less frequently reported.

**Case description:**

We report the case of a 47-year-old female diagnosed with an isocitrate dehydrogenase (IDH)-mutant World Health Organization Grade 3 astrocytoma located in the medial orbitofrontal cortex. The patient underwent gross total resection via a minimally invasive supraorbital trans-eyebrow craniotomy. Early postoperative MRI performed within 72 hours confirmed gross total resection without residual contrast-enhancing tumor. Histopathological examination demonstrated microvascular proliferation consistent with Grade 3 astrocytoma, and molecular analysis confirmed IDH mutation. The patient subsequently received standard adjuvant radiochemotherapy with temozolomide. Follow-up demonstrated preserved neurological function and high cosmetic satisfaction.

**Conclusion:**

This case illustrates that, in carefully selected patients, the supraorbital keyhole approach can achieve oncologically adequate resection of medial orbitofrontal IDH-mutant high-grade gliomas while minimizing surgical morbidity and optimizing cosmetic outcomes.

## Introduction

High-grade gliomas (HGGs) represent the most aggressive primary tumors of the central nervous system and are associated with rapid progression and poor prognosis. Combined radiotherapy and temozolomide-based chemotherapy remains the standard adjuvant treatment in high-grade gliomas (1). Achieving maximal safe resection is a critical component of management, as the extent of tumor removal has been consistently correlated with improved survival outcomes (2, 3).

Minimally invasive cranial approaches have evolved to reduce surgical morbidity while preserving oncological adequacy. The supraorbital trans-eyebrow approach has been widely described as an alternative corridor to the anterior cranial fossa (4, 5). Beyond technical feasibility, cosmetic and patient-reported outcomes have also been increasingly emphasized, with several studies documenting favorable aesthetic satisfaction following eyebrow incisions (6–8).

Despite the growing body of literature on supraorbital craniotomy, detailed reports focusing specifically on molecularly characterized IDH-mutant Grade 3 astrocytomas located in the medial orbitofrontal region remain limited. We therefore present a case highlighting oncological adequacy, surgical strategy, and functional and cosmetic outcomes achieved through a minimally invasive supraorbital keyhole approach.

## Case description

Patient Information and Clinical Findings: A 47-year-old female was admitted with an eight-month history of intermittent headaches, dizziness, and generalized weakness. The headaches were described as wave-like, predominantly in the evening, and partially responsive to analgesics. There was no history of seizures or prior neurological disease. General somatic status was stable.

Neurological examination revealed no focal deficits. The patient was fully alert and oriented, with intact cranial nerves, preserved motor and sensory function, normal coordination, and stable gait. No signs of intracranial hypertension or meningeal irritation were observed.

Diagnostic Imaging: Magnetic resonance imaging (MRI) with contrast revealed a 22 × 17 × 12 mm lesion located on the medial surface of the left orbitofrontal cortex. The lesion demonstrated heterogeneous internal architecture on T2-weighted and FLAIR sequences, with central areas suggestive of necrosis. Contrast-enhanced T1-weighted images showed irregular ring-like peripheral enhancement and moderate surrounding vasogenic edema. No midline shift or significant mass effect was present (**Figures 1A–F**).

**Figure 1:**
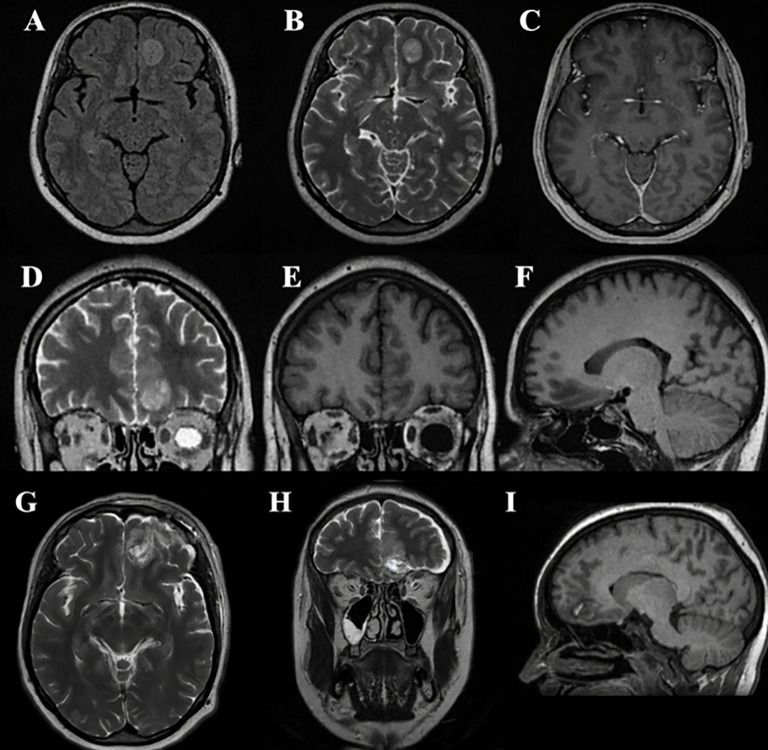
*Preoperative and postoperative magnetic resonance imaging (MRI).* Preoperative MRI demonstrates a 22 × 17 × 12 mm heterogeneous lesion located on the medial surface of the left orbitofrontal cortex. Axial FLAIR-weighted **(A)**, axial T2-weighted **(B)**, axial contrast-enhanced T1-weighted **(C)**, coronal T2-weighted **(D)**, coronal T1-weighted **(E)**, and sagittal T1-weighted **(F)** images show a centrally necrotic lesion with irregular peripheral contrast enhancement and moderate surrounding vasogenic edema. Early postoperative contrast-enhanced T1-weighted MRI obtained within 72 hours after surgery demonstrates gross total resection without residual enhancement or significant peritumoral edema on axial **(G)**, coronal **(H)**, and sagittal **(I)** views.

Diagnostic Assessment and Differential Diagnosis: Based on imaging characteristics, a high-grade glial tumor was suspected. The primary differential diagnosis included glioblastoma (WHO Grade 4), anaplastic astrocytoma (WHO Grade 3), and oligodendroglioma (WHO Grade 2–3). The absence of extensive mass effect and the preservation of relatively well-defined margins favored Grade 3 astrocytoma over glioblastoma. Lack of calcifications and absence of 1p/19q codeletion on subsequent molecular testing made oligodendroglioma unlikely. Brain abscess and metastatic disease were excluded based on clinical and radiological findings. Given the imaging profile and clinical presentation, surgical resection was indicated to establish definitive diagnosis and achieve maximal safe tumor removal.

Therapeutic Intervention: A minimally invasive supraorbital trans-eyebrow craniotomy was performed under microscopic guidance. The patient was positioned supine with slight head rotation and fixation in a three-point Mayfield holder. A 3 cm incision was made along the natural eyebrow crease, and a supraorbital bone window approximately 2 cm in diameter was created. Intraoperative neuronavigation was used throughout the procedure to confirm the surgical trajectory, define lesion margins, and minimize unnecessary cortical exposure through the limited supraorbital corridor.

Following dural opening, a limited corticotomy was performed. Dynamic brain relaxation techniques and minimal fixed retraction were employed to reduce focal pressure on surrounding brain tissue during tumor exposure and resection. At a depth of approximately 3 cm, a soft, grayish, vascular tumor was identified. Stepwise microsurgical resection was achieved using suction and bipolar coagulation. A macroscopically complete resection consistent with gross total resection (GTR) was accomplished. Estimated resection volume was approximately 2.5 cm³. The dura was reconstructed using a synthetic graft with watertight closure. The bone flap was repositioned and fixed. The procedure duration was 2 hours and 30 minutes, with estimated blood loss of 150 mL. No intraoperative or postoperative complications occurred (**Figure 2**).

**Figure 2:**
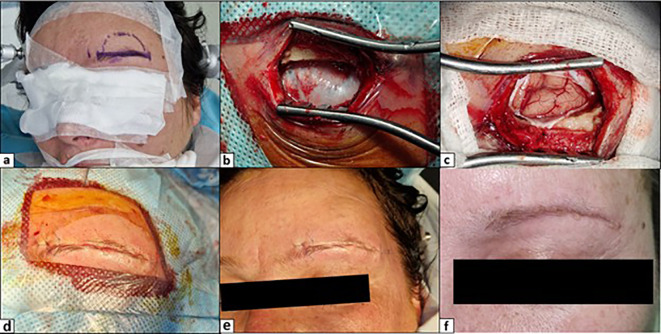
*Stages of the supraorbital trans-eyebrow (keyhole) approach for frontal lobe tumor resection.* **(a)** Preoperative marking: planned skin incision along the projection of the left supraorbital arch; head secured in Mayfield holder, eyes protected with sterile dressings; **(b)** Soft tissue dissection and exposure of the orbital roof; creation of a bony window; **(c)** Dural opening with an arcuate incision; brain tissue visualized with gentle microsurgical exposure of the operative corridor; **(d)** Closure phase: layered suturing and cosmetic skin closure; **(e)** Early postoperative outcome: minimal edema, clean cosmetic incision; **(f)** Late postoperative outcome: satisfactory aesthetic result, with a scarcely visible scar anatomically aligned with the natural eyebrow crease.

Histopathological findings: Histopathological examination revealed astrocytic tumor tissue with architectural heterogeneity, mitotic activity, and microvascular proliferation. No necrosis sufficient to reclassify the tumor as Grade 4 was observed. Immunohistochemistry demonstrated strong diffuse glial fibrillary acidic protein (GFAP) expression. Molecular testing confirmed IDH1 p.R132H mutation, with absence of IDH2 mutation and no 1p/19q codeletion. The final diagnosis was astrocytoma, IDH-mutant, WHO Grade 3 (**Figure 3**).

**Figure 3:**
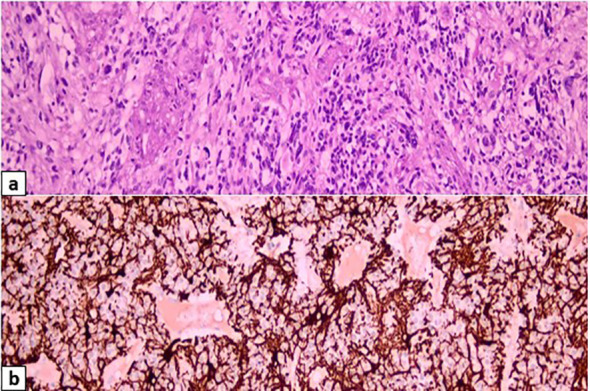
Histopathological and immunohistochemical features of the resected tumor. **(a)** H&E staining (×200) reveals a heterogeneous tumor structure with honeycomb-like areas, optically clear cytoplasm, perinuclear halos, and frequent mitotic figures. **(b)** Immunohistochemical staining shows diffuse positivity for GFAP, indicating glial differentiation.

Adjuvant Therapy and Follow-Up: The patient was discharged on postoperative day 7 in satisfactory condition (**Table 1**). Early postoperative MRI performed within 72 hours after surgery confirmed gross total resection without residual contrast-enhancing tumor (**Figures 1 G–I**). Adjuvant treatment included radiotherapy (total dose 54 Gy in 2 Gy fractions) combined with temozolomide administered at 75 mg/m² during radiotherapy followed by adjuvant cycles at 150–200 mg/m².

**Table 1:** Timeline of clinical presentation, diagnostic evaluation, surgical treatment, adjuvant therapy, and follow-up.

Time	Event
Month - 8	Onset of intermittent headaches and dizziness
Month –3	Progressive increase in headache frequency
Day 0	Hospital admission and neurological examination
Day 1	MRI with contrast revealing medial orbitofrontal lesion
Day 2	Supraorbital keyhole surgical resection
Postoperative Day 1	Neurologically intact; early symptom improvement
Postoperative Day 7	Discharge in satisfactory condition
Week 3	Initiation of adjuvant radiochemotherapy
Month 5	Follow-up MRI confirming no recurrence

At 5-month follow-up, contrast-enhanced MRI demonstrated no evidence of residual tumor, recurrence, or peritumoral edema (**Figures 4A–C**). The patient remained neurologically intact. Cosmetic evaluation using the Patient and Observer Scar Assessment Scale (POSAS) revealed excellent results (patient score 6/60; observer score 7/55), with the scar minimally visible along the natural eyebrow line.

**Figure 4:**
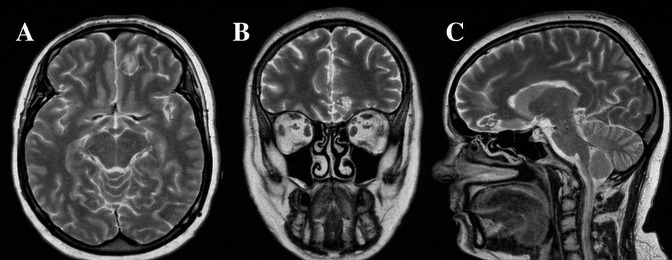
Postoperative magnetic resonance imaging (MRI). Postoperative contrast-enhanced T1-weighted MRI obtained at 5-month follow-up demonstrates complete tumor resection without evidence of residual enhancement or peritumoral edema on axial **(A)**, coronal **(B)**, and sagittal **(C)** views.

## Discussion

Maximal safe resection remains a cornerstone in the management of diffuse gliomas. While traditional large craniotomies have been widely utilized, minimally invasive keyhole approaches have evolved as alternative surgical corridors (9–12).

The supraorbital trans-eyebrow approach has been extensively described for anterior skull base and parasellar lesions (11–14). Over the past two decades, technical refinements and expanded indications have broadened its application to intraaxial tumors (15–18). Several operative series have demonstrated the feasibility of supraorbital craniotomy for supratentorial lesions (19–21), including selected high-grade gliomas of the frontobasal region (22).

However, most published studies include heterogeneous patient populations and mixed tumor types. Dedicated analyses of molecularly characterized IDH-mutant Grade 3 astrocytomas treated via a pure supraorbital corridor remain limited. In contrast to broader supraorbital series (9, 11, 14, 19), the present report focuses specifically on a medial orbitofrontal intraaxial glioma with defined molecular status.

A key concern when adopting minimally invasive approaches for high-grade gliomas is whether adequate oncological exposure can be achieved without increasing the risk of incomplete resection. Previous technical descriptions have emphasized the importance of patient selection, tumor size, and anatomical constraints (10, 23–26). In appropriately selected cases with limited tumor volume and absence of significant mass effect, the supraorbital approach may provide sufficient visualization and surgical maneuverability. Ideal candidates for the supraorbital keyhole approach include patients with small to moderate tumor volume, predominantly medial or anterior skull base localization, limited lateral extension, absence of severe mass effect, and manageable perifocal edema. Lesions with extensive lateral infiltration, deep extension beyond the operative corridor, or significant intracranial hypertension may be less suitable for this minimally invasive approach.

It is important to acknowledge that minimal craniotomy does not universally equate to reduced brain retraction. Comparative analyses between pterional and supraorbital approaches have shown that surgical corridor limitations must be weighed against exposure benefits (27). In cases of extensive edema or deep lateral extension, wider craniotomies may remain preferable. Thus, anatomical suitability and careful preoperative planning are critical determinants of success. Compared with conventional pterional or bifrontal craniotomies, the supraorbital approach offers reduced soft tissue disruption, smaller skin incision, and potentially shorter postoperative recovery. However, larger craniotomies may provide broader multidirectional exposure in tumors with extensive lateral or deep extension, emphasizing the importance of individualized surgical planning and careful assessment of factors associated with surgical morbidity (28).

From a technical perspective, refinement of microsurgical technique and adjunctive technologies have contributed to improved safety and precision (23, 25, 29). The ability to achieve gross total resection in the present case without neurological deficit supports the feasibility of this approach in selected orbitofrontal lesions. Other minimally invasive strategies, including endoscopic endonasal and eyebrow-assisted variants, have also been explored for anterior skull base lesions (30). Nevertheless, the supraorbital trans-eyebrow approach provides a direct transcranial corridor with familiar microsurgical anatomy and flexibility for intraaxial tumor resection.

Cosmetic and patient-reported outcomes are increasingly recognized as relevant components of neurosurgical care. Several series have documented high levels of cosmetic satisfaction following supraorbital craniotomy via eyebrow incision (7, 21, 31, 32). Although aesthetic considerations should not supersede oncological priorities, they contribute meaningfully to overall patient satisfaction and quality of life.

### Limitations

As this is a single-case report with a relatively short follow-up period, conclusions regarding long-term oncological control and progression-free survival should be interpreted with caution. In addition, the applicability of this approach to larger, more infiltrative, or laterally extending tumors remains uncertain. Molecular analyses for MGMT promoter methylation and CDKN2A/B homozygous deletion were not available and therefore could not be included in the integrated characterization of the tumor. Formal neurocognitive and quality-of-life assessments were not performed because the patient had no preoperative neurological or cognitive deficits and remained neurologically intact throughout follow-up. However, the lack of standardized measures limits objective assessment of functional recovery beyond routine clinical evaluation and cosmetic outcomes. Further studies involving larger patient cohorts and longer follow-up are needed to better define patient selection criteria and evaluate the long-term outcomes of the supraorbital keyhole approach for high-grade gliomas.

### Take-home message

In carefully selected patients with small medial orbitofrontal gliomas, the supraorbital keyhole approach can provide adequate surgical exposure while maintaining functional and cosmetic outcomes. Thorough anatomical evaluation and strict patient selection remain essential to ensure oncological safety.

## Patient perspective

The patient reported significant relief of headaches in the early postoperative period and expressed satisfaction with the overall treatment process. She particularly noted the minimal visibility of the surgical scar and the absence of noticeable facial asymmetry. At follow-up, the patient reported preserved cognitive function and return to normal daily activities without limitations. She expressed confidence in the treatment outcome and satisfaction with both the functional and cosmetic results.

## Data Availability

The original contributions presented in the study are included in the article/supplementary material. Further inquiries can be directed to the corresponding author.
